# Climate change and non-migration — exploring the role of place relations in rural and coastal Bangladesh

**DOI:** 10.1007/s11111-022-00402-3

**Published:** 2022-05-21

**Authors:** M. M. Golam Rabbani, Matthew Cotton, Richard Friend

**Affiliations:** 1 Department of Environment and Geography, University of York, York, UK; 2 School of Social Sciences, Humanities and Law, Teesside University, Middlesbrough, UK

**Keywords:** Place relations, Place obduracy, Non-migration, Climate adaptation, Bangladesh

## Abstract

Of growing research and policy interest are the experiences of people living under conditions of climate change–induced environmental stress, which either are unable to migrate (sometimes described as a ‘trapped population’) or are seemingly unwilling to do so (sometimes described as the ‘voluntarily immobile’). This paper problematises and expands upon these binary categories: examining the complex dimensionality of non-migration as a form of place relations, explored through qualitative study of rural and coastal Bangladeshi communities. Through 60 semi-structured interviews of individuals from four communities in the Kalapara region, the analysis proffers four qualitatively derived and inter-related dimensions of voluntary and involuntary non-migration framed as a form of place relations. These four dimensions concern the following: (1) livelihood opportunities, (2) place obduracy, (3) risk perceptions, and (4) social-structural constraints, with the interplay between these elements explaining diverse non-migratory experiences. In our analysis, ‘place obduracy’ is introduced as a concept to describe the differential speed of environmental change and socio-cultural adaptation responses to explain non-migratory experiences. Our discussion provides insight into how to best support non-migrant people’s adaptive capacity in the face of growing climate emergency.

## Introduction — climate change, migration, and non-migration

Of growing concern for migration scholars and adaptation practitioners are the intensifying challenges of forced migration resulting from global conflict, social unrest, and environmental change (Miller, 2019; Owain & Maslin, 2018; UNFCCC, 2017). The United Nations High Commissioner for Refugees reports that the number of people forced to migrate worldwide has almost doubled in the past decade, reaching a current estimate of 79.5 m; forced migration now affects more than one per cent of humanity (UNHCR, 2020; IPCC PSB AR6, 2021). With the escalating threat of climate emergency, *environmental stress* factors are of growing global research and policy concern. It behoves policy authorities to respond to migratory pressures driven by environmental stress, including those that arise as a result of sea level rise, encroaching salination, extreme weather events, temperature and precipitation changes (Hugo, 1996; Hunter, 2005; McLeman, 2009; Black et al., 2011; Kniveton et al., 2011; Chen & Mueller, 2019), fire (Paton et al., 2008), biodiversity loss, flood, and disruption to ecosystem services (Adams & Adger, 2013; Adie, 2020; Bonaiuto et al., 2016; Vaske & Kobrin, 2001). The World Bank estimates that up to 143 million *climate migrants* could emerge in three regions (Latin America, sub-Saharan Africa, and Southeast Asia) in response to these myriad threats by 2050 (Rigaud et al., 2018). However, it must be noted that it is difficult to delineate climate-related drivers of migration from other socio-political and economic factors. As such, predictions and population estimates have become contentious issues in debates over environmental migration (Gemenne, 2011).

Despite difficulties in establishing precise population estimates and drivers of environmental migration, clearly coastal and rural communities within developing nations are at growing risk of a migratory response to the climate change impacts that threaten social welfare and regional economic and political security. Migration is often framed as an adaptive response to environmental stress (Singh & Basu, 2020) – in essence a coping strategy to reduce mortality, morbidity, and livelihood loss associated with climate change. Migration, however, receives significantly more attention than *non-migration* in academic and policy circles. The so-called stayers (non-migrants) require much greater research scrutiny (Hjälm, 2013). There is an emergent research agenda exploring the experiences of those that are labelled as either *voluntarily immobile* (Wiegel et al., 2021) or *involuntarily immobile* in response to climate-induced risks (Zickgraf, 2019; Ayeb-Karlsson et al., 2018; Mallick & Schanze, 2020). The latter are often described as a ‘trapped’ population (Black & Collyer, 2014).

The seemingly binary categories of voluntary and involuntary non-migration belie the complexity of socio-environmental and economic conditions, cultural ties, household livelihood strategies, and other internal and external factors that lead to non-migratory responses. Non-migration is a complex area of social research, embedded within a matrix of socio-cultural values, expectations, and place relations that inform individual, household, and community-scale migratory decisions (Wiegel et al., 2021). This matrix links migratory and non-migratory decision-making with a host of related socio-cultural and physical environmental factors including cultural conditions, economic structures, and livelihood opportunities (Adams, 2016; Logan et al., 2016; Ayeb-Karlsson et al., 2018; Zickgraf, 2019; Mallick & Schanze, 2020); community histories and oral traditions (Nunn & Campbell, 2020); religious and spiritual beliefs and practices, economic opportunities, family ties, and social responsibilities (Cohen & Sirkeci, 2011); and adaptive capacity and perceptions of environmental threat. Non-migration research therefore necessitates an understanding of social complexity, sense-making, and place-making.

In this paper, we aim to problematise and explore the experiences of non-migrants living under conditions of both acute and long-term climate-induced environmental stress through qualitative exploration of at-risk communities in Bangladesh. We examine the features and processes of local sense-making that occur towards climate-induced threats and migratory opportunities (Nawrotzki & DeWaard, 2018), including locally specific social representations of nature and human-nature relations (Wiegel et al., 2021), attachments to family and work (Kelman et al., 2019), and how these translate into migratory and non-migratory conditions for at-risk communities (Farbotko et al., 2016). Through qualitative analysis, we explore the dialectic of voluntary and involuntary non-migration phenomena through the lens of *place relations* – contributing to the emerging literatures on the social and economic geography of climate change adaption responses within vulnerable communities.

## Human-place relations and migration/non-migration

Much of the existing research on non-migratory decision-making falls within the discipline of social psychology – exploring the push and pull factors that influence the range of social choices available to migrating/non-migrating peoples. However, critics have argued that reducing migration/non-migration to *decision-making* as a form of rational choice obscures the broader social-structural, cultural, aspirational, and adaptive capacity factors that influence migratory outcomes and experiences (Diener & Hagen, 2020; Farbotko & McMichael, 2019). Though environmental stress is an important factor, environmental risks alone are a weak predictor of migratory responses (Massey et al., 2010). It is the interaction between environmental conditions and issues such as land ownership, inheritance and household wealth, land use rights, the strength of social networks, ties of social capital, as well as identities of *translocality* (Sakdapolrak et al., 2016) – including mobility, circulation and spatial interconnectedness – that provide context and significance to environmental risk responses (Mallick et al., 2021).

Environmental push factors interact with social structures and relations, and so, migratory/non-migratory decisions can be understood as outcomes from complex livelihood strategies that households adopt in order to increase their coping capacity with change and uncertainty (De Haas, 2010; Logan et al., 2016; Maxmillan, 2016; Aniah et al., 2019; Biswas & Mallick, 2020). Multiple environmental factors influence this capacity (Rustad et al., 2019; Jackson et al., 2020), but the deeper dynamics between concurrent environmental, economic, demographic, political, and social changes are necessarily place-based, temporal, and interactive (De Haas, 2014; Jahan et al., 2015; Hoogendoorn et al., 2020). Place and place-making are therefore essential socio-material conditions of migratory/non-migratory outcomes (Brehm et al., 2013; Simoni & Floress, 2015).

Place and place-making appear across multiple academic literatures. There are five primary concepts discussed in the literatures of geography, environmental psychology, urban studies, and sociology that encompass individual and community relations with place. We summarise the key concepts here as:

1. *Place dependence* – the functional provision of particular resources that support human activities (Williams & Vaske, 2003).
2. *Place attachment* – a psychological process (Adams, 2016; De Dominicis et al., 2015; Devine-Wright, 2013; Vaske & Kobrin, 2001) that posits an emotional place-bond between individuals and their environment (Adie, 2020; Tuan, 1974).
3. *Place identity* – an affective counterpart to place dependence, through which sentiments around self-identification are formed (Farnum et al., 2005).
4. *Place utility* (Adams & Adger, 2013; Baker, 1982) which in a migration context focuses on satisfaction/dissatisfaction as a component of migration decision-making processes (Baker, 1982; Haer et al., 2017).
5. *Sense of place* (Tapsuwan et al., 2011) is a spatial and subjective phenomenon that encompasses the emotional and spiritual aspects of human-place relations. This sense of place evolves over time (Nicolosi & Corbett, 2018)*.*

In this paper, we also posit a novel sixth component of place relations – *place obduracy* – to describe the conditions under which the rate and scale of sociocultural and socioeconomic change lags behind concurrent changes in environmental conditions. We explain place obduracy as a type of maladaptation, in which local place relations create a barrier to migration and, as such, increase the associated risks to people experiencing environmental stress. The *place obduracy* concept is a new theoretical framework for studying non-migration emerging from our qualitative data analysis and is thus discussed in greater detail in the “Results” section below.

From a research perspective, we embrace holistic, inclusive, and intersectional understandings of the relationship between place, capacity, and aspiration (Nicolosi & Corbett, 2018) that cut across the six conceptual categories mentioned above. Thus, we use the terminology of *place relations* throughout. *Place relations* conceptually embodies a multidimensional approach: accommodating a range of both voluntary and non-voluntary relationships, including attachments, cultural barriers, and resource constraints (Adams, 2016). Place relations form the primary conceptual framework within this paper and our empirical study uses this framework to synthesise new insight into the combination of environmental, social, economic, psychological, and place-based components of non-migratory decision-making under conditions of escalating environmental risks from climate change (Staller, 2008; Ardoin et al., 2012; O’Donnell, 2019). In doing so, we draw upon qualitative community stakeholder interviews in the critical case of rural and coastal Bangladesh, as residents of this country experience both acute and chronic climatic change–induced environmental stress and complex community adaptive and migratory/non-migratory responses.

## Country background — environmental migration and non-migration in Bangladesh

Over the last decade, Bangladesh has undergone substantial economic and structural development. Exports of ready-made garments, remittances, and agricultural produce, alongside digital technology growth (including internet access and mobile banking), have accelerated Bangladeshi citizen access to financial markets and global investment (Khatun et al., 2021). Though the national picture shows a positive story of economic modernisation and growth, this is unevenly geographically distributed. For primarily rural and coastal climate-vulnerable regions and communities, current and future disaster impacts severely reduce economic potential, and this perpetuates a vicious cycle of poverty (Monirul Alam et al., 2017; Rigaud et al., 2018; Matin et al., 2018).

The differential economic and environmental risk experiences of urban and rural/coastal Bangladeshi peoples make the country a critical case of early observable evidence of forced climate-induced migration and non-migration (Kelman, 2018). The risks of river and coastal erosion, repeatedly inundated homes and farmlands, food scarcity, and economic and non-economic losses create an urgent environmental and development crisis. It is estimated that 80% of farmers reported that crop and livestock production are suffering from unseasonable rain, limited availability of surface water, and depletion of groundwater (Aryal et al., 2020). Summer temperatures are also rising above levels for productive rice cultivation (Ministry of Foreign Affairs of the Netherlands, 2018; Mojid, 2020). Collectively, these changes will negatively impact agricultural GDP by an estimated 3.1% each year (World Bank, 2010), threatening the food security of subsistence farmers most keenly. Coastal fisheries in Bangladesh are also affected (Khanam, 2017; Rahman et al., 2020). Low-lying lands in the tidal flood-prone regions are becoming increasingly saline, forcing the disappearance of native fish species (Miah et al., 2020), alongside growing vulnerability of aquaculture infrastructure.

The potential adaptive responses to climate impacts from farmers and coastal fishers in the Bay of Bengal are further stymied by other pollution factors and inadequate resource management (Habib et al., 2014). These collective factors lead to estimated economic losses of 1.7 billion USD by 2050 (Das et al., 2020). For fishers, staying longer onshore due to the increased frequency and magnitudes of extreme weather events reduces their earning capacity and increases reliance upon local money lenders (Uddin et al., 2019), compounding the financial vulnerability of small-scale food producers while deepening their dependence on exploitative patron-client relations (Wood, 2003). Disaster-affected farmers and fishers move towards non-farm employment as a coping strategy to tackle short-term reductions in total household income (Eskander et al., 2016). Non-farm livelihoods in the context of coastal Bangladesh are small enterprises such as corner shops, pop-up stalls, and carts in the coastal markets, or for those with no other viable assets, selling labour in the form of pulling vans, driving rickshaws, and bike-cabbing. Though changes in livelihood strategy locally provide a short-term solution to environmental stress-induced vulnerability, factors such as coastal erosion force markets to move further inland, and storm surges demolish road and retail infrastructure for long periods. Moving merchandise to safety during cyclones is therefore a challenge for entrepreneurs. These short-term changes in livelihood strategy are therefore unlikely to reduce long-term climate vulnerability. Sadly, multihazard maps for coastal Bangladesh warn that the existing vulnerabilities of coastal communities are likely to increase by 2050 and beyond (Jahan et al., 2016; Ministry of Foreign Affairs of the Netherlands, 2018; Kirezci et al., 2020). These growing challenges to the life and livelihoods of coastal Bangladeshis have direct impacts on human-place relationships. Many regions of Bangladesh have become critical case studies for understanding migratory and non-migratory responses under environmental, economic, and social stress, and such case studies have additional global significance for similarly climate vulnerable parts of the world where local economies are dominated by small-scale farming and fishing livelihoods.

## Case study — Kalapara region

**Fig. 1:**
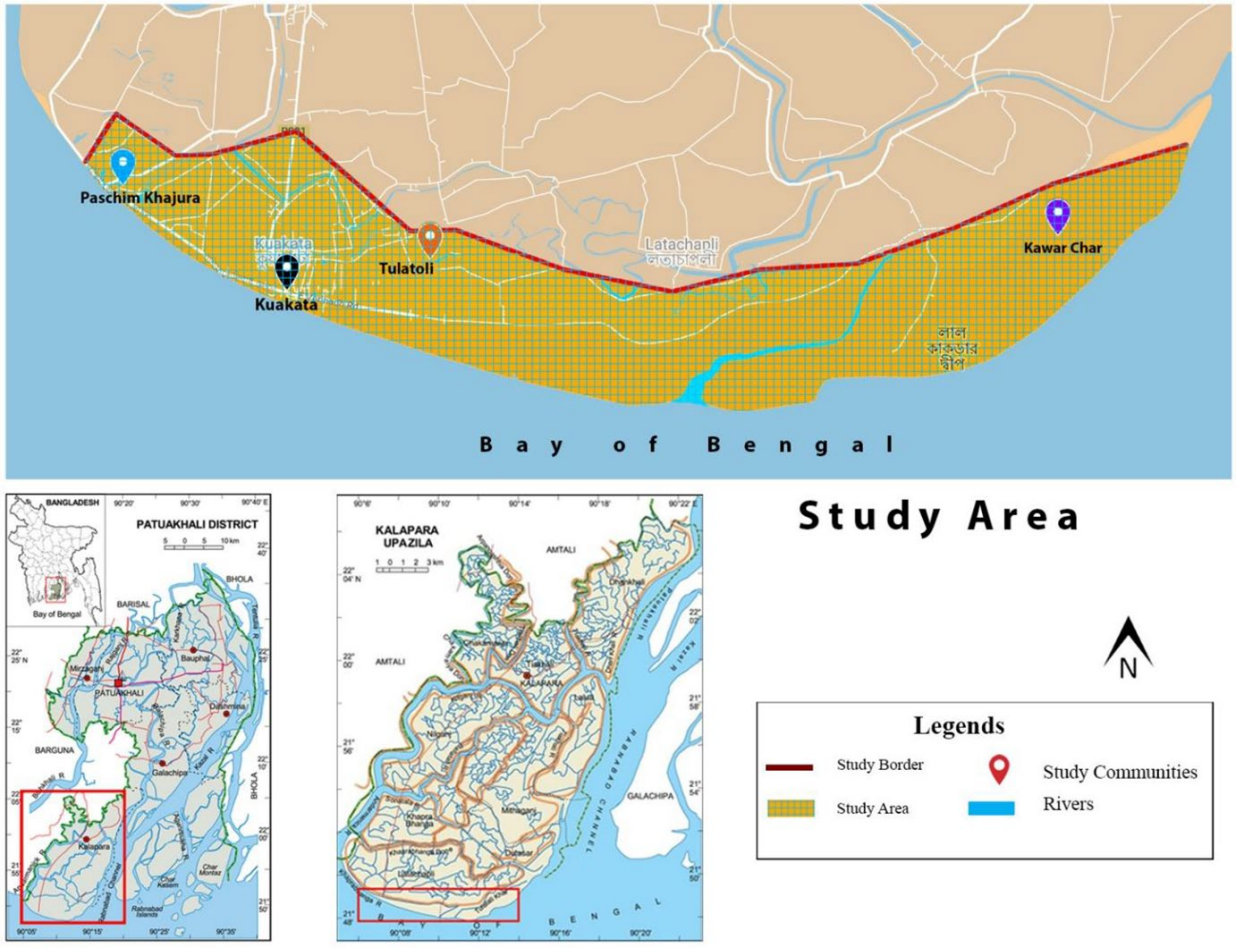
Study site in Kalapara, Patuakhali, Bangladesh

In exploring migratory and non-migratory conditions in Bangladesh, our empirical study uses a place-based strategy for participant sampling, examining individuals’ experiences and responses to environmental stress, migratory, and livelihood decisions through qualitative interviews with 60 participants in the sub-district (Upazila) coastal town, Kalapara, in Bangladesh, as shown in Fig. 1.

Kalapara Upazila was selected as a case region due to its diverse coastal economy, including farming paddy, fishing, and tourism-based businesses, combined with growing environmental stress related to cyclones, storm surges, fluvial-tide flooding, coastal erosion, and encroaching salinity (Ahamed et al., 2012; CEGIS & GoB, 2013; Hasan & Akter, 2019). Recent regional evaluation also shows the growing number of environmentally induced displaced people living there (Shamsuddoha et al., 2012; Bernzen et al., 2019). Together, these factors make Kalapara a place of research importance for environmental migration/non-migration conditions and experiences.

Geographically, the region lies approximately 200 miles south of Dhaka and includes Kuakata – a long sandy beach along the Bay of Bengal. The 2011 Bangladesh census shows Kalapara has 31,324 households and a population of 174,921. Four communities were selected across the coastal area to provide a sample of 60 interviewees with backgrounds across a range of livelihood sources (farming, fishing, and small businesses – see Table 1).

**Table 1:** Community sites, participant numbers and dominant livelihoods

**Community name**	**Number of participants**	**Major livelihood sources**
**Paschim Khajura**	3 couples, 10 males, and 1 female	Fishermen and farmers
**Tulatoli**	4 couples, 9 males, and 3 females	Farmers and daily labourers
**Kawar Char**	2 couples, 10 males, and 3 females	Fishermen and farmers
**Kuakata**	15 males	Businessmen and farmers

## Methods

The researchers used in-depth semi-structured interviews to explore local experiences of disaster impacts, the socio-environmental context of their work and home life, and their decision-making regarding migration and non-migration from and within the region. Attention was paid to those with locally situated livelihoods so that shared experiences (such as from the impacts of the same extreme weather events) could be captured across the interviews (Adeola, 2007). However, it is important to note that though extreme weather events affect the whole community, not all participants across each livelihood category experience these events in the same way; each has a different adaptive capacity in the face of such threats (Fischer & Chhatre, 2016). Adaptive capacity also differs across ethnic and religious groups (Sen et al., 2020), and so, local diversity was taken into account, including respondents from minority Rakhine communities.

Within the non-random sample, we aimed to recruit decision-makers across low, medium, and high-income households, with respondents represented in each livelihood category. We note that decision-making at the household scale is dominated by wage-earners. This created a problem of gatekeeping, one that inevitably skews our respondent sample towards the perspectives of male wage-earners. Specific efforts were made to include women in the interview process, though cultural barriers concerning household role and interaction with male interviewers limited women’s participation. The study tried to include all decision-makers of a household interviewed together whenever possible to optimise data quality, as adaptive decisions are usually joint efforts of the members in a household. As such, we discuss the findings at the scale of the household, though interviews were with individual respondents (Hossain et al., 2017).

All interviews were recorded in the respondents’ home. No incentives were offered. Written consent was obtained. Interviews were conducted in Bangla and were recorded audio-visually. Bangla transcripts were translated into English. The lead author is a bilingual speaker. English transcriptions were coded using computer-aided qualitative data analysis software NVivo™ using thematic analysis, whereby a coding framework was established to capture relational and expressive values through a sequential process of description, interpretation, and explanation of qualitative data. This process established a multilevel framework of coding – from top-level thematic coding to establish the context and production processes of broader themes, subsequently followed by a more detailed examination of utterances to better understand the psychological, socio-cultural, and political-economic context and to produce an interpretive framework (Baxter & Eyles, 1997; Braun & Clarke, 2006). Emergent themes form a four-part top-level coding framework used to structure the results below, each representing a dimension of human-place relationships built through the interpretive analysis of coded utterances.

## Results

Through inductive qualitative analysis, we identify four themes across a continuum of place relations dialectically linking issues of structure and agency, migratory, and non-migratory conditions (Chouinard, 1997) discussed below:

1. Livelihood opportunities
2. Place obduracy
3. Risk perceptions
4. Social-structural constraints

### Livelihood opportunities

Livelihood opportunities are understandably the primary socio-economic context within which place relations are defined, with most participants volunteering this as a principal factor in their utterances. At the time of data collection (pre-Covid-19 pandemic, early 2020), the significant livelihood opportunities available locally were farming, coastal fishing, small businesses, and informal employment/day labouring. Livelihood opportunities were increasing at the point of data collection due to government support for transforming Kuakata into a tourist attraction. Most new opportunities were in the hospitality industry: either working in hotels, as tourist guides, or bike cabbing. However, it must be noted that we would expect livelihood diversification strategies that rely upon tourism to be adversely impacted by Covid-19 in the short-medium term (Islam, 2021).

Smallholder and subsistence farmers’ perspectives differ from those of lease-taking farmers of small holdings and day labourers on larger farms. Subsistence farmers have a smaller asset base and usually insufficient savings or ties of indebtedness to relocate. Landless farmers are arguably the most vulnerable, given their limited asset base and most of their skills are specific to farm labour. Land-owning farmers with sufficient capital or access to credit to invest reported tend towards agricultural diversification – either saline-tolerant rice, crops resistant to extreme weather events, higher value crops such as mango and coconut, and cattle/poultry farming. Some also consider fishing as a viable option depending on their skills, asset base, and social networks. For example, one male interviewee identifies as a middle-class farmer and fisherman in Kawar Char. His family of eight members has three working members. Collectively, the family has repeated experience of crop loss, as he states:

The embankment collapses easily and floods the farmland. This island is full of farmers. If they can’t make enough money from farming, then it is a big problem. People go fishing, but it does not make enough. But if farming goes well, people can make a good profit.

As shown by this utterance, the *potential* for profit remains a decisive motivating factor – given that crop loss is not limited to one specific region, agricultural vulnerability from specific weather events is insufficient to force migration for subsistence farmers. As with farming, boat owners and day labourers have very different livelihood strategies. Within our study, most of the day-labourer fishers live in government-built huts along the coast or their ancestral land. Some also owned small patches of farming lands where rice and vegetables are grown, and others owned hens, goats, and cows. However, most household income stems from fishing labour. Most fishing opportunities go to physically fit men, with very few opportunities for women. Traditionally, women work as homemakers or less commonly as day labourers drying fish or farm-working. A male coastal fisher, who self-identifies as lower-middle-class, explains that for day labourers, there is increasing diversification of livelihood types and that flexibility under conditions of uncertainty is essential, stating:

When it [catch] was low, the fishermen had to work outside. For example, they had to work as labourers, harvesting crops on others’ lands, pottery, whatever skills they had. Whatever work they found, they did to carry on. Recently, as the catch has gone up, we are better now. Here is good for living. Because the demand for fish in here is high, people here can also do other things, for example, farming, business, etc... The availability of different [livelihoods] helps me to keep going.

This utterance describes new income-opportunities and limited local economic market diversification, enhancing livelihood outcomes and contributing to improved adaptive capacity manifested as better economic wellbeing (Kelly & Adger, 2000; Peña-Lévano et al., 2019). But it also indicates the dependence on labour markets over which they have very little influence. Livelihood opportunity is geographically rooted for vulnerable households. For example, certain livelihood activities’ skills and experience influence people to carry on living in the same location despite environmental risks. Another male coastal fisher states:

This is our fishing place. I was born here. Where else would I go? What will I do there? I am good at fishing. I know where to get more fish around the coasts of Kuakata. You don’t get fish everywhere in the sea.

Livelihood opportunities for small business owners are also a vital dimension of place relations. Around a quarter of interview participants owned small businesses, usually small shops on the coasts on the seaward side of the embankment. These shops are highly vulnerable to storm surges, erosion, and sea-level rise. One respondent owns a small souvenir shop in the coastal market that is his only source of income. He described his livelihood opportunities and environmental risks as:

My souvenir shop is only good in the tourist’s places. And most tourist places are on the coasts. So, the risks are everywhere.

More than half of the participants reported multiple sources of income. Recent economic development initiatives have expanded employment opportunities for local communities. Some had started working for construction companies who used to be working in farming, fishing, or as day labourers before. Tourism-based self-employment, i.e. bike cabbing, photography, and selling street foods, has further diversified income streams. These new opportunities are a decisive motivating factor in explaining the conditions of non-migration. The perceived threshold of environmental risk is not yet high enough for most participants to ignore these livelihood opportunities available, even though those risks are rapidly increasing in severity and frequency in the case study location.

### Place obduracy

The place relations that root people within local economic opportunities, known social and environmental risks are complex and multidimensional. Despite many sufferings from insecure livelihoods and exposure to health and safety risks from extreme weather events, the socio-psychological bonds with which respondents create a sense of place (of where they are born, grow up, work, and live) are a powerful rooting factor. To explain these relationships, we draw upon the aforementioned components of sense of place, place dependence, place identity, and place attachment (Mulvaney et al., 2020), while adding *place obduracy* as another key dimension of place relations, based upon emergent perspectives from interviewees (as described below).

Despite the experience of having lost crops, farmlands, livestock, and sadly even members of the family due to extreme weather events, place identities, dependencies, and attachments remain strong. Place identity refers to the psycho-social bonds that link an individual to their community. Locality, culture, language, memory, and kinship harmonize to create a sense of home, one that links familial bonds and intensifies the sense of community as an established ‘in-group’, and this stretches across social classes and income levels. For example, a self-identified middle-class Rakhine farmer has multiple small patches of farmlands, a family of five members, and lives in Dariaramkhola Para. The household has multiple experiences of crop loss due to flooding and cyclones. Yet he explains the emotional bonds with the place he lives alongside recognition of limited livelihood opportunities elsewhere:

Where would I go leaving this place? I was born and grew up here. My ancestors have lived here. If we go somewhere, it won’t be any easier for us.

A farmer respondent built his hut on a patch of land allocated by the local government in Kawar Char. He and his son work as day labourers in between fishing and selling fish products in the local market. He describes his household as poor and expresses his experience of environmental conditions: “[there are] *no limits to disasters here*”. However, he identifies the place where he lives accordingly:

We leave our homes during disasters and go to the cyclone shelter. When the disaster is gone, we come back home and start all over again … Brother, we cannot go far. Who is going to look after my children? Where will we go? My birthplace is here. My father and my grandfather were born here.

The psycho-social and cultural ties to place, such as family history and ancestral memory, blend together with recognition of livelihood constraints and practical considerations, such as childcare, material constraints such as land or homeownership and renting, schooling, and caring responsibilities. Notably, these two elements are discussed in concert with one another. For example, a fisherman living in Abasan, the government-built shelter in Khajura describes his dependency as:

Because we don’t have any land, either to farm or to live. We could not send our children to school. I have always been poor. The only work I have learnt is fishing. We all are fishermen here.

It is in this way that place identity from cultural ties links to place dependence from the material constraints that trap the poorest people into routines of low paid work and (in this case subsidized) housing, which in turn, then embeds families within social networks that are place-based and relatively static. Even when new places provide features and conditions that support specific economic goals, i.e. fertile farmlands, availability of fish in the sea, demand for labour, etc., these are insufficient to disrupt the sense of place attachment felt by respondents.

We find that when a place’s physical characteristics change rapidly under evolving environmental conditions (making a place unsafe), the relationship between place identity, dependence, and attachment does not change at the same speed. The sense of place persists within the community imagination even when the nature of that place changes rapidly due to environmental stress. We describe this phenomenon as *place obduracy*, related to livelihood opportunities that rapidly encroach upon communities due to climate change are not met with social adaptations to reduce vulnerability. In a similar manner to *sociotechnical obduracy* discussed in other fields of urban geography (Cresswell & Hoskins, 2008; Hommels, 2005), place obduracy describes the conditions under which human-place relations adapt too slowly to meet the (new and emerging) needs and circumstances of a community. Place obduracy establishes a non-migratory decision-context. In this context, participants who experience place obduracy will commonly have a specific skill set, such as extensive fishing experience, but little formal school education or support to adapt through alternative skill training. We found that it is difficult for many respondents to think about picking up new skills or moving away to an environmentally ‘safer’ region. However, the relationship between skills, economic opportunity, livelihood strategy, and migration differs across class boundaries. Indeed, for the lowest paid respondents with the least material assets (particularly land, property, and capital), there is a more substantial degree of labour migration because this has become a feature of their household livelihood strategies due to their limited asset base. One respondent, a day labourer with no farmland, living in the ancestral home with three members describes how he works someone else’s farmlands or goes fishing in someone else’s boat; however, he states:

I want to live here with my family. If I want to leave, I probably can. However, I do not want to leave.

There is evidence here of a perceived capacity to adapt the livelihood strategy through migration, but the non-migratory decision is bound up in respondents’ place identity. Respondents imagine themselves as coastal or rural people with corresponding locally embedded skills, cultural practices, and preferences. Place identity for people of rural Bangladesh is unique from their urban counterparts, and this identity remains stable across class and income boundaries. Therefore, we can explain place obduracy as a position in which the environmental risk threat has not yet exceeded a threshold or tipping point to trigger a migratory response – the sense of place and livelihood constraints that respondents experience continues to anchor them to specific geographic spaces and communities, even when the threat to life, wellbeing, and livelihood from environmental risk is growing over time. It is self-reflection and growing awareness of imminent risks – the sense of uncertainty and unease regarding livelihood futures and the need for diversification strategies through new skills and income opportunities – that reveal early signs of disruption to place obduracy and hence a motivation for migration either now or in the future.

### Risk perceptions

Interviewees in Kalapara experienced multiple and overlapping environmental hazards, including cyclones, storm surges, salinity intrusion and coastal erosion. However, there remains a stark difference between scientific assessment of regional risk factors and the expressed perception of these risks by interviewees. Direct experience of hazards is not a clear indicator of a strong desire to migrate. For some, the thought of migration has not consciously entered their decision-making. Instead, they are familiar with coping with disasters, for example, by taking safe shelter and then returning when the cyclone subsides, and the flood waters recede. Responses to environmental hazards are mediated through the *nature* of the risk (Lechowska, 2018) and its *influences* (Satter & Cheung, 2019). It is a combination of the physical and emotional experience of these two factors that play an essential role in the formation of risk perceptions (Bronfman et al., 2020). However, this process is complex and differentially distributed across age, gender, community status, education, household demographics, and class structures (Clar, 2019; Lee, 1966). The intersection of risk perception and migratory decision-making is not as straightforward as a push or pull mechanism. For example, a female shopkeeper in Kuakata and farmer near Tulatoli describes her aspiration towards migration as:

Yes, [laughs]. When storm hits, crops get damaged, then I think of leaving. When the disasters is over, then… [she laughs again, implying a change of mind].

The reasons for the exclusion of migration as an adaptation response are explained by two principal mediating factors – on the one hand, familiarity, and normalisation of the landscape of risk, and on the other, the perception of risk management responsibility. Also of note here is the use of humour to normalise and diminish the sense of risk (Parkhill et al., 2011).

One participant, a male subsistence farmer and fisher described the risks to his farm and house as:

If Allah wants to keep someone alive, who can kill! He has created us. He is the one who will look after us. This is why we stay here, confidently. The environment is good here. I haven’t thought of going anywhere. Rich people think of buying land in safer places, not people like us.

We see that religious beliefs, ethical norms, value systems, and specific socio-cultural structures and institutions influence risk perception (Renn & Rohrmann, 2000; Lee et al., 2015). Interviewees commonly expressed risks in fatalistic ways, drawing on religious convictions – with utterances that display an acceptance of hardship as part of the human condition (Jahan et al., 2015). Another male small business owner, who has farmlands from Kuakata bazaar and self-identifies as poor, similarly states:

Yes, we want to raise her [daughter] here because this is her ancestral land too. The rest is the Will of Allah. If the government can keep the embankment secure, everybody here will be able to live peacefully.

These statements speak to *fatalistic* responses to risk management, to use the terminology of ‘grid-group’ theory (Douglas & Wildavsky, 1982; Spickard, 1989), familiar to social contexts in which religious belief mediates local place-based knowledge and experience (Jahan et al., 2015). A supernatural explanation of control over seemingly external hazards such as cyclones or other life-threatening or livelihood-disrupting events is a means to cognitively resolve the sense of powerlessness felt in the face of harm and conversely strengthen the sense of personal agency. What is novel here is the introduction of a social class element – that hierarchical and egalitarian risk responses are blended with the fatalistic description – safety through migration is for the rich, and safety through religious observance is for the poor. Yet as the second statement shows, this is mixed with a concern that the Bangladeshi government must step in to protect the vulnerable through hard infrastructure risk mitigation responses. Aspirations towards migration are therefore grounded and contextualised in broader webs of religion, class, income, and trust-in-government relationships. Risk management at the household level is not a simple matter of moving away from the site of risk to protect personal safety or property. There is little sense of *personal* agency primarily due to these other cognitive, sociocultural, and structural-material factors that disempower the individual to act in the face of climate risk. As one fisher describes:

I went four years back. I used to drive in Dhaka. My mother died, and I had to come back. I used to earn a lot but could not save. I thought I would stay with my father and give him a hand. I have a fishing boat and net too.

The quote illustrates the social context of the male-dominated household in which men are expected to be primary income providers. The gendered work structure entrenches specific livelihood strategies in rural Bangladesh. In this case, the quoted male interviewee is solely responsible for family income. Economic opportunity stimulates rural-to-urban migration though the relatively higher cost of urban living outweighs any financial security benefit gained from employment. Changes in family and life circumstances (such as bereavement) disrupt this fragile livelihood strategy. Migratory decision-making is balanced against a combination of personal, familial network ties and environmental risks from fishing and farming. Thus, even when migration is experienced, it is not the primary or dominant adaptation response given the place and community-focused loci of decision-making that respondents experienced.

### Social-structural constraints

As our interviewees express, environmental migration (Foresight, 2011; Logan et al., 2016; Ayeb-Karlsson et al., 2018) in response to temperature change or changes in the disaster profile of a place is insufficient to explain migratory adaptation responses. Under conditions of extreme deprivation, migration is constrained by the lack of multiple capitals (including financial and social) necessary to relocate (Zickgraf, 2019). We assess these elements as forms of social-structural constraint (Bakewell, 2010) that limit the decision-making capacity of the individual or household. We find that, contrary to expectation, social-structural and material constraints to migration do not always correspond directly with financial poverty. Multidimensional economic, social, political, and personal health factors, directly and indirectly influenced by slow and fast onset environmental drivers, all play a role. Though we make no claims to demographic proportional representation in the interview sample, it is nonetheless interesting to note that less than one third of participants mentioned that they intended to migrate but could not do so due to one or more external constraining factors. Through thematic analysis, we break down social-structural constraints into four sub-thematic categories of emergent utterances concerning: *insufficient means*, *socio-demographic constraints*, *poor governance*, and *geographical constraints,* which leads to what is often described as the ‘trapped population’ problem–where people want to leave but lack the agency or capacity to do so.

Respondents commonly discuss the condition of insufficient means. For example, a male souvenir shopkeeper at the coast of Kauata Bazar self-describes himself as poor and has four other household members. When a cyclone hits the region, it is weeks before he can reopen his shop, meaning that he has considered migrating away from the area. However, a lack of financial assets provides a critical barrier to migration. He states:

I have to have money to think that way [moving to a safer place]. I can’t afford that. So, I keep that aspiration [of migration] in me.

As climate change exacerbates the frequency and severity of extreme weather events, coastal livelihoods become untenable due to the prolonged periods of closure and economic inactivity that result. Where respondents have small or unstable social networks, there is little recourse to formal livelihood support systems (such as bank loans or government grants). This leads to reliance upon local money lenders that are often private business owners. For example, participants mentioned boat owners that set out loans in exchange for an agreement to work in fishing. Debt then creates indentured servitude, severely limiting their agency in many aspects of social life, including migratory decision-making and the effective marketing of personal skills (the indentured personal may not have fishing skills, for example), in a “Faustian Bargain” (Wood, 2003).

The conditional constraint of insufficient means works across familial networks. The case of a father of three sons who does not work regularly anymore illustrates the following point. His sons work as a tailor, labourer, and priest in a local mosque in Kolatoli respectively. They consider themselves poor. The father had an aspiration to send one of his sons to work abroad to send remittances home. However, he never had enough money to diversify their existing livelihoods. He describes the phenomenon as:

I have already grown old. But my sons and I, we are in a combined family. My son is a priest here in Kuakata. What will he do outside? Migration needs money. We don’t have that money.

Age, gender, and disability in at-risk households can reduce their respective coping capacities, simultaneously creating place-based constraints. The means and agency to migrate are thus linked to socio-demographic constraints, specifically those relating to financial and social capital and the flexibility and stability of social and knowledge networks. To illustrate, a day labourer respondent has a family of four members in Kolatoli. He explains the social context uncertainties that he faces:

Let’s say I go somewhere new. I am not known to anybody there. I will have to work to live there. It’s not like that that I go somewhere, buy a business. On the other hand, everybody knows me since I was a child. Everybody calls me if they need someone to work.

Familiarity, networks of association, and bonding capital create conditions of livelihood stability. The expressed concern for a lack of social networks in other places then becomes a barrier to regional outmigration. In Bangladesh, place-based social capital is a significant factor in employment in both rural and urban areas. Those with substantial social capital across a place-based network use this to build resilience (Matin et al., 2018).

The capacity to migrate is not just a matter of financial assets but how an individual can utilise those assets within broader a broader context of social, political, and institutional constraints. The challenges of weak political governance at national-to-local scales result in inefficient risk management and climate maladaptation. Examples of weak governance can be seen in land management and administrative practices. For example, a male subsistence rice farmer in Tolatoli self-describes as having a small amount of private land and considers himself poor. The participant describes poor governance and corruption limiting his ability to take migratory decisions:

Increased land price has created many problems for us. Even if you have records in the land registry, you can’t have the land. The politically powerful people in society are taking control of the land by producing false documents and using local goons.

Mobility for rural people requires stable land management systems and governance, clear tenure and ownership rights, rights of access, and support mechanisms for financing land use and making productive use of land. Under conditions of what is reported as corrupt local governance, the breakdown in land management systems becomes a key constraint on the migratory agency. However, state intervention in the provision of early warning systems and emergency shelters has been considerably more effective, leading to significant reductions in disaster-related fatalities and injuries across the country. Yet such successes have unintended consequences of creating a false sense of security in the long-term efficacy of state-led disaster risk reduction (in line with White (1954) concept of the ‘levee effect’, whereby disaster protection measures act as a perverse incentive to remain or settle in high-risk locations). It remains to be seen whether it will be the failure in such measures that ultimately incentivises local people to migrate, and if so, at what scale of severity such failure might occur.

Geographic location shapes patterns of human-place relationships through access to natural resources and livelihood provision. For the poorest people, areas of abundant natural resources are increasingly prone to cyclones and storm surges, for example, a smallholding farmer in Tolatuli farms chillies, butternut squash, and bitter vegetables. Extreme precipitation or wind will destroy the entire crop. In 2019, this farmer lost his chilli farm entirely along with thousands of other farmers. In response to why he still lives in the region, he responded:

I neither know any business nor can get a job. I could not study. My parents died when I was young. I had to work to keep the family going… To survive, what other work can we do? If we want to live, we have to work [on my farmlands].

Limitations in education qualifications, broad skills suitable for other economic sectors, and caring responsibilities keep this participant trapped in the same livelihood strategy despite concern that it is no longer tenable. For the participant to fully adapt to the risks created by environmental stressors requires a complex set of support mechanisms. It is necessary for policy authorities to provide livelihood diversification through skills training, support mechanisms for family networks, and land governance that allows for smoother transactions in changing land ownership.

## Conclusions

This study contributes to a broader understanding of non-migratory decision-making in the face of intensifying climate change and illustrates the influence of material assets, social relations, and subjective and psychological dimensions of place relations for climate-vulnerable households. In qualitative evaluation of rural and coastal Bangladeshi experiences of climate change, we find four overarching themes of *livelihood opportunities, place obduracy, environmental risk perceptions,* and *social-structural constraints*. The first three in this list represent core aspects of social agency: the capacity of the individual or household to define their livelihood, cultural observance, and familial networks within a place, even when these are threatened by external environmental risks. The last theme, structural constraints, represents the inability to make choices and/or the inhibition of decision-making capacity. These overarching themes explore the multidimensionality of place relations – that there is a dialectical relationship between structural and agential factors in defining place relations in contrast to a binary distinction between voluntary and involuntary migratory and non-migratory conditions.

We argue that migration and non-migration are dependent upon the sensitivity of the affected households to environmental change mediated through place relations. The vulnerabilities experienced and their adaptive capacity are primarily a function of what we term *place obduracy* – describing the speed at which socio-cultural and economic change within a place can adapt to changing environmental conditions. Place obduracy as a subset of place relations provides an novel conceptual framework through which to explain why and how maladaptation occurs, the cultural values and local political economy in which migratory and non-migratory conditions occur, how decisions are taken, how such decisions are constrained by social-structural relations, and the timing of such decisions in the face of growing climate change–induced environmental stress. In short, where social-institutional, risk perception, and livelihood constraints on migratory agency lag behind changing environmental conditions, this causes further injustice to occur within environmentally stressed households, and this provides grounding for future research into non-migration under conditions of climate emergency.

For many interviewees, the lack of assets and supportive social relations outside of their current community setting provide substantive socio-cultural and material barriers to migration. Current locally based livelihood opportunities, however precarious, are considered economically viable – they still allow residents to remain in place. This is, however, unlikely to be tenable in the future, as climatic change outpaces obdurate social adaptation. Thus, while the focus of our research is on non-migratory decision-making of individuals and households, it is also important to consider the point at which the state accepts the necessity of planned retreat (migration) as the only viable option for vulnerable populations in the face of intensifying climate change. Adaptation responses that emphasise hard infrastructure protection for coastal regions, local job creation programmes in tourism, and rehousing schemes for coastal residents may prove ineffective or maladaptive and thus prolong settlement in locations that will be no longer viable. Under conditions of place obduracy, as a form of place relations, residents may continue to support in-place adaptive measures such as this, even when they no longer provide sufficient safety, wellbeing, and livelihood protection. Adaptation planning and disaster risk management, therefore, require a careful, socio-culturally sensitive development response: understanding that decision-making at the lowest level, addressing social-structural inequalities, a robust understanding of climate risks, and uncertainty, transparency, and accountability, collaboration among a range of stakeholder groups is crucial to optimise the informed adaptation practices that manifest through stable place relations, or else, the challenge of internal and cross-border displaced population will remain a persistent threat to the poorest at-risk households in Bangladesh and other climate change–stressed regions globally.
